# Costs of delivering human papillomavirus vaccination in Tanzania

**DOI:** 10.1093/eurpub/ckac131.393

**Published:** 2022-10-25

**Authors:** V Struckmann, V Stephani, A Hsiao, D Mbbando, J Changalucha, K Baisley, A Levin, R Hutubessy, D Watson-Jones, W Quentin

**Affiliations:** 1 TU Berlin, Berlin, Germany; 2 HelloBetter, Berlin, Germany; 3 Mwanza Intervention Trials Unit, Mwanza, Tanzania; 4 LSHTM, London, UK; 5 Levin & Morgan, Bethesda, USA; 6 Immunization, Vaccines and Biologicals Department, WHO, Geneva, Switzerland

## Abstract

**Background:**

Cervical cancer caused by human papillomavirus (HPV) is the most frequent cancer in women in many low-income countries.Tanzania implemented a national HPV vaccination program in 2018 using a two-dose quadrivalent HPV vaccine. This study aimed to (1) estimate financial and economic costs of a two-dose vaccination program based on experiences with the national vaccination program, (2) estimate costs of a one-dose vaccination schedule to enable future cost-effectiveness analyses, and (3) assess the effect of alternative assumptions for future vaccination coverage rates on estimated costs of vaccination.

**Methods:**

The WHO Cervical Cancer Prevention and Control Costing (C4P) tool was used to estimate the incremental costs of the national vaccination programme from the perspective of the Tanzanian government using data collected via surveys, workshops, and interviews with local stakeholders. Deterministic sensitivity analyses were performed to estimate the effect of alternative assumptions for coverage rates and delivery strategies and to assess the impact of a potential one-dose vaccination schedule.

**Results:**

The total financial and economic costs were US$10,117,455 and US$45,683,204, respectively, at a financial cost of $5.17 per two-dose fully immunized girl (FIG), and an economic cost of $23.34 per FIG. Under the assumption of a one-dose vaccination schedule, costs per FIG would reduce to financial costs of $2.51 and economic costs of $12.18.

**Conclusions:**

The overall cost of Tanzania’s HPV vaccination program was lower per vaccinee than previous demonstration projects in the region suggest. These data provide important baseline data for Tanzania’s HPV vaccination program to date and may serve as a guide for improving coverage going forward. The findings may also aid in the prioritization of funding for countries that have not yet added HPV vaccines to their routine immunizations.

**Key messages:**

• If a single dose regimen were found to be as effective as a two-dose series, it would result in significant cost savings as well as an increase in the number of girls that could be reached.

• School-based vaccinations resulted in the lowest price per fully immunized girl, but other settings are needed to achieve equitable high coverage of HPV vaccination in Tanzania.

